# *SCRIP*ting a Path to Scholarship: How Student Journals Reduce Barriers to Publication

**DOI:** 10.1007/s40670-025-02286-y

**Published:** 2025-02-04

**Authors:** Niraj Vyas, Sonia Lobo

**Affiliations:** Department of Medical Education, Geisinger Commonwealth School of Medicine, 525 Pine Street, Scranton, PA 18509 USA

**Keywords:** Research, Publication, Student journals, Publishing, Scholarship

## Abstract

Early initiation of research activities among medical students is associated with later academic success and improved scientific productivity; however, barriers to publication in mainstream journals may deter students from disseminating their scholarly work. We sought to determine the impact of *Scholarly Research in Progress* (*SCRIP*), a student journal, on students’ level of experience, knowledge, or practice related to writing and publication, writing apprehension, and scholarly productivity. Students from Geisinger Commonwealth School of Medicine were surveyed via Qualtrics to assess their experience and motivation to publish, determine publication rates, and better understand barriers to publishing practices. Data was reported using means and standard deviations for ranked questions. One hundred sixteen participants responded, and 77 completed surveys were included in the analysis. Participants who had previously published in *SCRIP* indicated they were more confident in writing manuscripts (*p* = 0.003), submitting articles (*p* = 0.002), navigating the publication process (*p* = 0.008), and navigating the peer-review process (*p* = 0.033) compared to those who had not previously published in *SCRIP.* *SCRIP*-published participants were also more likely to place a high value on publishing research in their careers (*p* = 0.028). There was no significant difference in the average number of total PubMed-indexed publications between *SCRIP*-published participants and non-*SCRIP*-published participants (*p* = 0.779). Overall, *SCRIP* positively impacted students’ attitude towards future publication and was associated with improved writing confidence. Reviewer feedback was well-received by students and helped to improve their research to a publishable level while supporting development of their scientific writing skills and confidence.

## Introduction

Early initiation of research activities among medical students is associated with later academic success and improved short- and long-term scientific productivity [1]. Furthermore, research experiences have become increasingly important to clinical practice, and the number of medical students performing research is growing [2, 3]; however, about 70% of research performed by medical students globally is unpublished [4]. Barriers to publication in PubMed-indexed medical or scientific journals may deter inexperienced student researchers from disseminating their scholarly work [5]. For example, PubMed-indexed journals often have stringent standards for the quality and rigor of the research they publish, and students may find it difficult to meet these high expectations, especially if they are new to the research process [6]. Balancing research with other academic responsibilities, such as coursework and exams, can be challenging for students. The time required to conduct, write, and revise research for publication can be substantial, and many students who are still in the early stages of their careers may not have experience in conducting or writing up research [5–7]. Having experienced mentors can significantly impact the quality of a student’s research; however, not all students have access to strong mentorship, which can hinder their ability to produce publishable work [5–8]. Additionally, most journals charge publication fees, which can be a barrier for students with limited financial resources or access to institutional support. The peer-review process can also be daunting for students, as receiving critical feedback and addressing reviewers’ comments require resilience and a willingness to revise and improve their work. Finally, students are particularly susceptible to targeting by predatory or pay-to-publish journals because they have limited research and publishing expertise [9].

Medical student journals serve as an accessible entry point for students to overcome some of these barriers and to publish their research [10]. Over 20 medical student journals exist worldwide, and they all share a common goal of fostering academic research and publishing among medical students [11]. Accordingly, they are an increasingly significant part of medical education, providing platforms for students to engage in research, publish their findings, and gain experience in academic writing and peer review. At Geisinger Commonwealth School of Medicine (Geisinger), we established the *Scholarly Research in Progress* (*SCRIP*) journal with the aim of fostering and disseminating student research and scholarship at our institution and providing a venue to support students’ development of scientific writing skills and confidence. The *SCRIP* journal publishes original work authored by medical and graduate students at Geisinger using a single-blind, peer-review process by Geisinger faculty; students can also serve as peer reviewers but are coupled with a Geisinger faculty mentor. All Geisinger students are invited to contribute their original research articles, reviews, short communications, letters, editorials, literature reviews, case reports, and cover art for consideration. *SCRIP* is published once annually in both print and online mediums and is edited and produced with the assistance of student editors engaged in the editorial and peer-review process. Since its launch in 2017, 187 student-authored articles have been published over seven issues. In this study, we sought to determine the impact of *SCRIP* on medical and graduate students’ level of experience, knowledge, or practice related to writing and publication, writing apprehension, and scholarly productivity.

## Materials and Methods

Geisinger medical and graduate students were surveyed to assess their experience and motivation to publish, to determine publication rates, and to better understand potential barriers to publishing practices. In total, the survey had between 9 and 18 questions. Branching logic was used to minimize redundance and facilitate completion. Questions with rank order or Likert scales were standardized to maintain consistency and minimize ambiguity. Members of the research team internally tested the survey to identify any issues with question wording, flow, or clarity and appropriate modifications were made through iterative discussions. The survey was administered over an 8-week period from late April to early June of 2021 to approximately 746 Geisinger medical and graduate students who were enrolled in the Doctor of Medicine or Master of Biomedical Sciences programs from 2017 to 2021 and included a mixture of current students and alumni. Potential participants were contacted by email containing a link to the online survey through a first contact attempt with one follow-up attempt. Students had 8 weeks to complete the survey. Incomplete questionnaires were excluded from the analysis. Surveys were conducted via Qualtrics and reported using means and standard deviations for all ranked questions. *G*-test of independence with William’s correction was performed for categorical data and two-sample *t*-test was performed for continuous data when to determine if publication in *SCRIP* impacted participants’ attitudes and confidence concerning writing and the publication process. The statistical data analysis was conducted in R 4.1.2 (The R Foundation for Statistical Computing). Student-authored research publications were retrieved from PubMed to determine publication rates. The study was approved by Geisinger’s Institutional Review Board.

## Results

Over the 8-week period, 116 participants responded to the survey. Missing survey information led to the exclusion of 39 respondents, yielding a final sample size of 77 completed surveys included in the analysis. Most respondents identified as female (53.2%) and white (68%) (Table 1). At the time of the survey, 59 participants (77%) were enrolled in the Geisinger medical degree program, and 20 participants (26%) had previously published manuscripts in *SCRIP*. Bivariate analysis of survey participants who had previously published in *SCRIP* indicated they were more confident in writing manuscripts (*p* = 0.003), submitting articles (*p* = 0.002), navigating the publication process (*p* = 0.008), and navigating the peer-review process (*p* = 0.033) compared to those who had not previously published in *SCRIP* (Table 2). *SCRIP*-published participants were also more likely to place a high value on publishing research in their careers (*p* = 0.028). There was not a significant difference in the average number of total PubMed-indexed publications between *SCRIP*-published participants and non-*SCRIP*-published participants (*p* = 0.779). There were also no significant differences in self-reported race and ethnicity between the two cohorts.

**Table 1:**
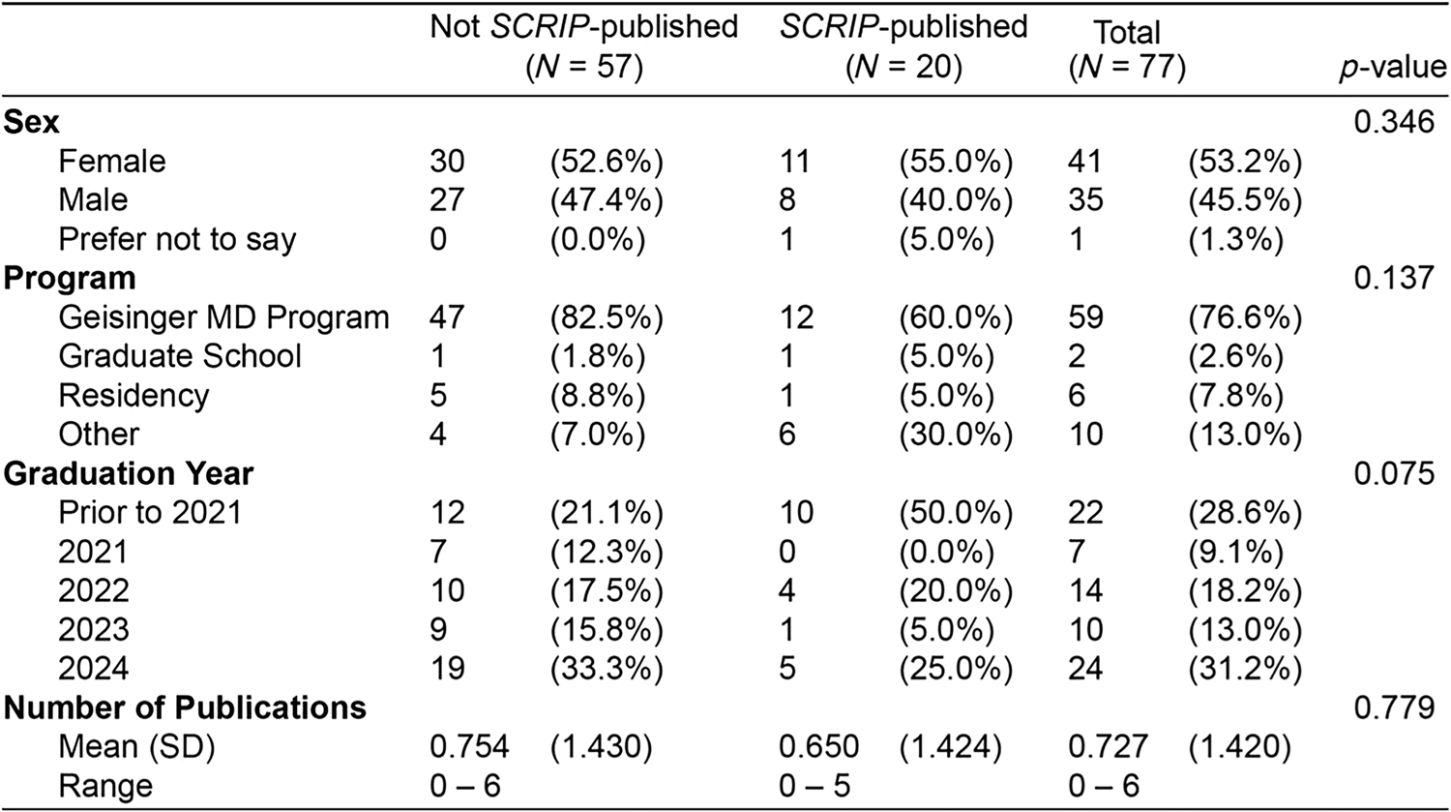
Socio-academic information of survey respondents

**Table 2:**
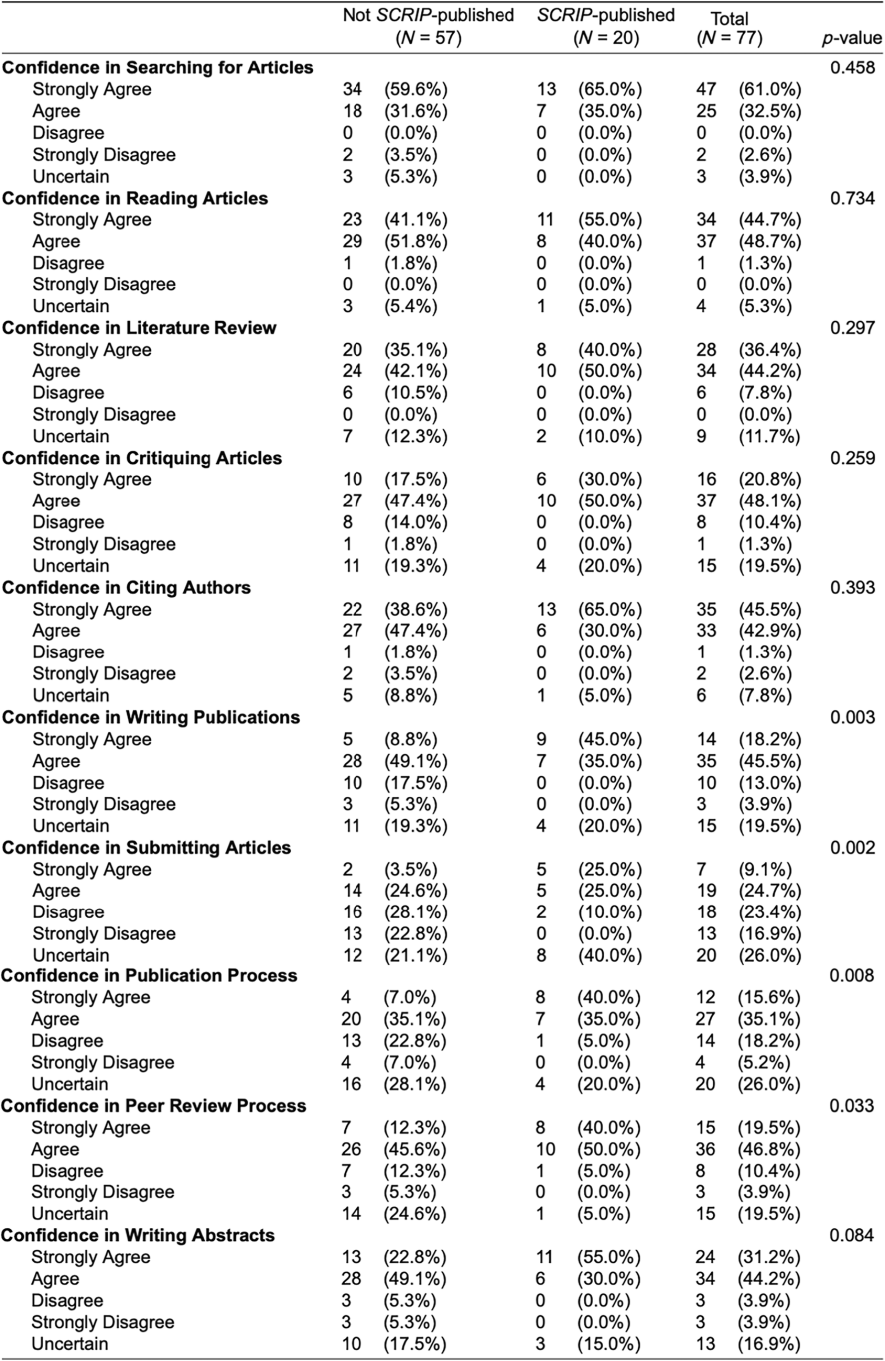

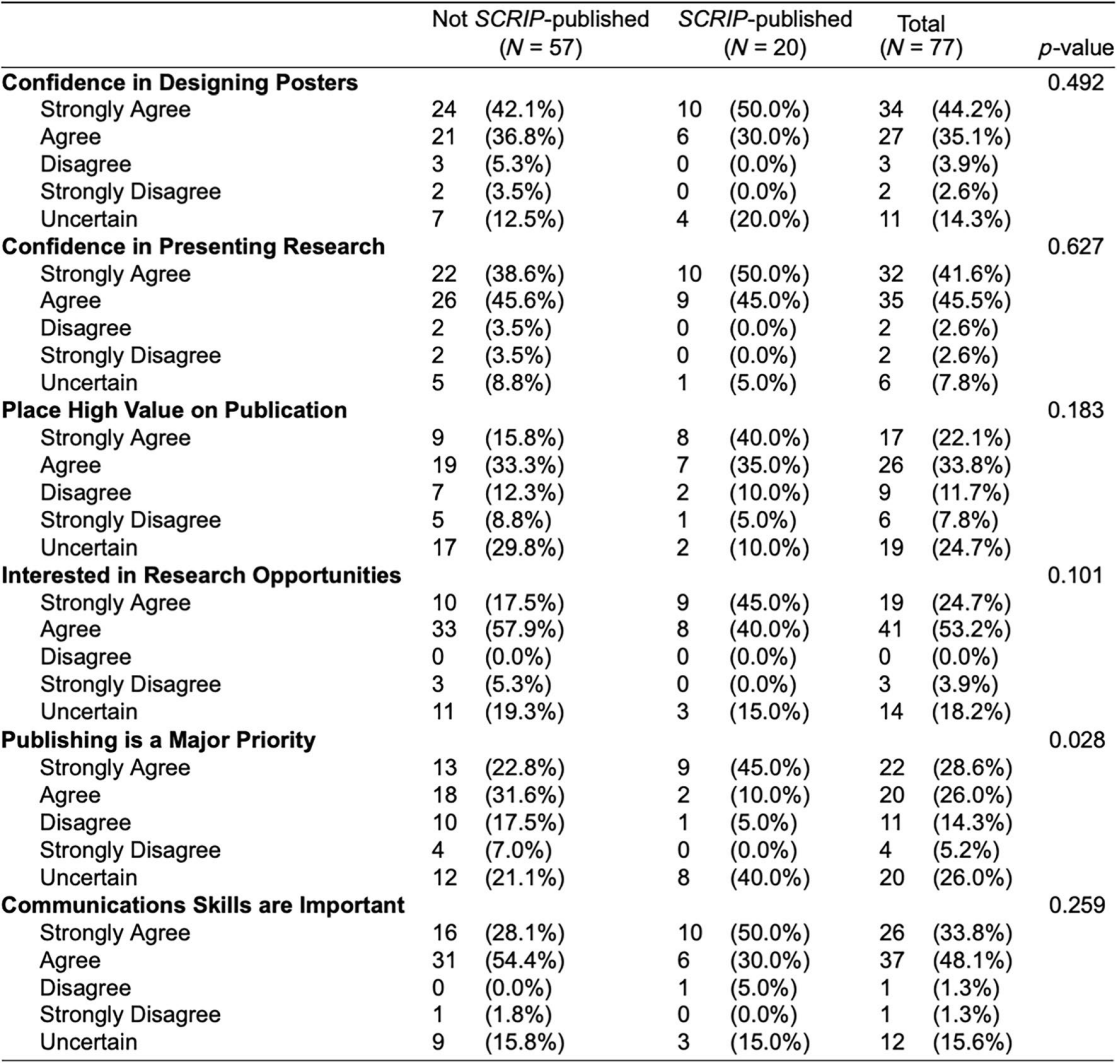
Bivariate analysis of survey participants who had previously published in *SCRIP* versus those who did not publish in *SCRIP*

Among *SCRIP*-published participants, 9 individuals (45%) said improving their career path was their main motivation to publish, while 5 individuals (25%) did so because they thought it was an important skill to learn (Table 3). Compared to expectations about publishing in *SCRIP*, most students’ experience was in line with expectations (45%) or better than expected (50%); moreover, students indicated that the peer-review process “moderately enhanced” (47%) or was one of the best parts (16%) of the publishing experience. When asked about submitting and/or publishing in *SCRIP* as a learning experience, 70% of *SCRIP*-published participants reported that it was fantastic (30%) or that they learned a lot (40%), while some were neutral about the experience (25%) or reported learning nothing (5%). Students indicated that publishing in *SCRIP* improved their ability to understand the publication process (90%), write up research for publication (80%), understand the peer-review process (75%), comprehend primary scientific literature (65%), write an article directed to other scientists (60%), and communicate science verbally (60%). Moreover, half of *SCRIP*-published participants reported that they were very likely to submit their manuscript for publication in a mainstream journal (40%) or that they had already done so (10%). When asked to rank the factors that prevented students from submitting an article to *SCRIP*, publishing in another journal was ranked most important (37%), followed by lack of time due to other commitments (21%), and then not having an opportunity to take part in research (14%).

**Table 3:**
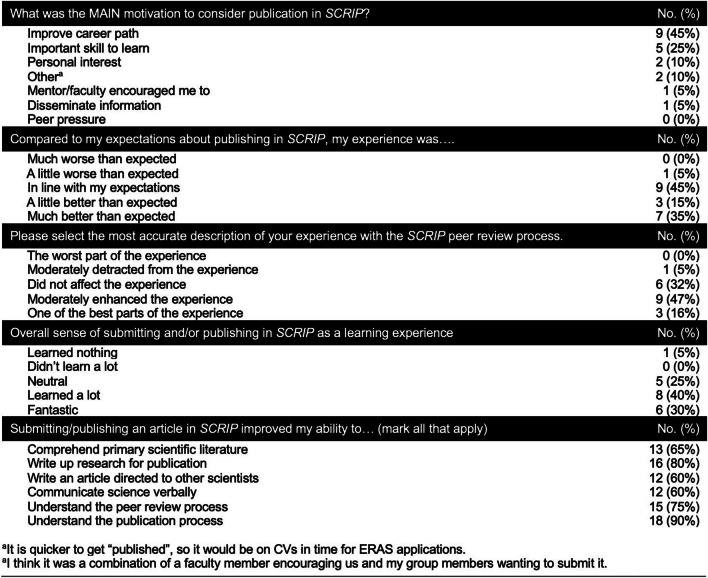
Motivation, expectations, experience, and self-reported improvement outcomes from 20 students who submitted and successfully published an article in *SCRIP*

## Discussion

The purpose of this study was to determine the impact of *SCRIP*, a student journal, on Geisinger students’ level of experience, knowledge, or practice related to writing and publication, writing apprehension, and scholarly productivity. A significant finding was that students who had previously published in *SCRIP* demonstrated higher confidence in various aspects of the publication process, including writing manuscripts, submitting articles, navigating the publication process, and the peer-review process. This increased confidence is likely attributable to the hands-on experience and mentorship provided by the journal and suggests that student journals like *SCRIP* are effective learning tools for developing essential academic skills. *SCRIP*-published participants also reported improvements in writing and research-related skills (Table 3); however, our survey did not directly measure whether their skills improved. Additionally, *SCRIP*-published participants placed a higher value on publishing research in their careers, underscoring the journal’s role in fostering a research-oriented mindset. No significant differences were observed in self-reported race and ethnicity between the two cohorts, indicating that the benefits of publishing in *SCRIP* are consistent across diverse demographic groups.

Interestingly, there was no significant difference in the average number of total PubMed-indexed publications between *SCRIP*-published and non-*SCRIP*-published participants, suggesting that while *SCRIP* enhances confidence and perceived value of research, it may not directly correlate with increased publication output in PubMed-indexed journals. Notably, the cross-sectional nature of this study captured data at a single point in time, making it difficult to infer causality or long-term productivity. Tracking *SCRIP*-published participants over an extended period could provide a more comprehensive understanding of the journal’s impact on future publication in PubMed-indexed journals. In addition, our search was limited to the PubMed database and did not account for articles indexed elsewhere or student authors whose names may have changed.

Motivations for publishing in *SCRIP* varied, with career improvement and skill acquisition being the primary drivers. Most students reported that their experience with *SCRIP* met or exceeded expectations, particularly valuing the peer-review process. This positive feedback highlights the journal’s effectiveness as a training medium for student scholarship. Students who chose not to publish in *SCRIP* reported that publishing in another journal was more important to them. While the survey did not ask students to specify the type of journal, they may have preferred to publish in a PubMed-indexed journal due to the presumed lower quality of student journals or their low visibility [11, 12]. Indeed, the *SCRIP* audience is narrower and Geisinger-specific; however, efforts are being made to enhance the online visibility of the journal and the discoverability of Geisinger student scholarship to the broader scientific community.

Opponents of peer-reviewed, medical student journals have argued that the peer-review process lacks transparency; therefore, medical students should not contribute their work. Further, they suggest that the scientific community is unlikely to be interested in reading articles published in “far less professional” journals [13]. Advocates for medical student journals believe they enhance scientific progress by training students in peer-review and writing standards from the very beginning of their careers in science and medicine [14]. In our experience, students have submitted their scholarly work to the *SCRIP* journal as a means of publishing smaller research studies or works in progress that later evolve into more comprehensive articles as their research matures. Since *SCRIP* authors retain the copyright to their publications, future iterations of their work can be submitted to PubMed-indexed journals with fewer barriers, including enhanced confidence and understanding of the publication and peer-review process. Notably, a recent study that examined long-term academic achievements of medical students who published in the *New Zealand Medical Student Journal* (*NZMSJ*) showed that these students were more likely to publish in PubMed-indexed journals, earn a PhD or other advanced academic degrees, and secure academic positions after graduation compared to their peers matched by gender, university, and graduation year [10]. Current investigation is underway to determine whether publication in *SCRIP* is associated with later academic success, including increased numbers of publications in PubMed-indexed journals post-graduation.

An unexplored aspect of this study is the potential of *SCRIP* to serve as a benchmark for written assignments in graduate and medical education. Some Geisinger professors already use *SCRIP* to guide the quality of systematic literature reviews in their graduate courses, aiming for publication-worthy results. Additionally, *SCRIP* is the final goal for a 4-year medical research honors program, transforming theses into peer-reviewed articles. The Biomedical Research Club, a student-led interest group at Geisinger, also promotes submitting manuscripts to *SCRIP* as the final deliverable for student research projects to increase their publishing potential. Similar journal-driven benchmarks have been reported; for example, at the University of British Columbia, progress towards submission of an original manuscript in an open-access online undergraduate research journal entitled *Undergraduate Journal of Experimental Microbiology and Immunology* (*UJEMI*), serves as the capstone for a course-based undergraduate research experience [15]. These practices could help provide a framework for other medical student journals to encourage publication as well as enhance the quality of submissions.

One limitation of our study is that our findings represent only a small cohort of students who published in *SCRIP*. Among this small cohort, the majority identified as white, female medical students which may not accurately represent the broader Geisinger student population and introduce bias, as the perspectives of other groups might be underrepresented. Additionally, participants who chose to respond to the survey might have had a particular interest or positive experience in publishing, which could skew the results; those with negative experiences or no interest might have opted out, leading to an overrepresentation of positive outcomes. Future studies could employ random sampling methods or encourage a larger, more diverse sample to enhance generalizability of the findings to other medical student journals and examine whether gender bias exists within our student authorship [16]. Prospectively measuring students’ motivations to publish and assessing the long-term impact of *SCRIP* publication experiences on academic success is a future goal. Overall, these findings highlight the significant role that student journals can play in the academic and professional development of medical students, suggesting that such platforms should be actively promoted and supported within medical education programs.

## Conclusions

Students who published in *SCRIP* are more confident in writing manuscripts, submitting articles, and navigating the research process, and are more likely to place a high value on publishing research in their careers. These findings underscore the multifaceted role that student journals can have in enhancing medical education, fostering a research-oriented mindset, and supporting the professional growth of future physicians. As the landscape of medical education evolves, student journals are poised to play an increasingly important role in bridging the gap between medical training and academic research. They provide an opportunity for medical students to engage in the publication process with fewer barriers than PubMed-indexed journals; further, they provide an enabling environment that facilitates the development of writing skills, writing confidence, and dissemination of generated scientific knowledge. Encouraging students to publish in student journals may also help them build a strong academic portfolio, making them more competitive for residency programs, future academic positions, and other career opportunities. With increasing pressure to produce scholarship for residency portfolios, student journals can also help medical students avoid serious consequences of publishing their work in predatory journals. Thus, student journals like *SCRIP* can serve as a valuable educational platform for trainee scholarship.

## Data Availability

Data is available upon request from the corresponding author.
